# The Combined Effect of Cold and Copper Stresses on the Proliferation and Transcriptional Response of *Listeria monocytogenes*

**DOI:** 10.3389/fmicb.2019.00612

**Published:** 2019-03-28

**Authors:** Ana María Quesille-Villalobos, Angel Parra, Felipe Maza, Paola Navarrete, Mauricio González, Mauricio Latorre, Magaly Toro, Angélica Reyes-Jara

**Affiliations:** ^1^Laboratorio de Microbiología y Probióticos, Instituto de Nutrición y Tecnología de los Alimentos (INTA), Universidad de Chile, Santiago, Chile; ^2^Millennium Nucleus in the Biology of Intestinal Microbiota, Santiago, Chile; ^3^Laboratorio de Bioinformática y Expresión Génica, Instituto de Nutrición y Tecnología de los Alimentos (INTA), Universidad de Chile, Santiago, Chile; ^4^FONDAP Center for Genome Regulation (CGR), Santiago, Chile; ^5^Mathomics, Center for Mathematical Modeling, Universidad de Chile, Santiago, Chile; ^6^Instituto de Ciencias de la Ingeniería, Universidad de O’Higgins, Rancagua, Chile

**Keywords:** *Listeria monocytogenes*, low temperature, copper, stress, global gene expression

## Abstract

*Listeria monocytogenes* is a foodborne pathogen that can cause severe disease in susceptible humans. This microorganism has the ability to adapt to hostile environmental conditions such as the low temperatures used by the food industry for controlling microorganisms. Bacteria are able to adjust their transcriptional response to adapt to stressful conditions in order to maintain cell homeostasis. Understanding the transcriptional response of *L. monocytogenes* to stressing conditions could be relevant to develop new strategies to control the pathogen. A possible alternative for controlling microorganisms in the food industry could be to use copper as an antimicrobial agent. The present study characterized three *L. monocytogenes* strains (List2-2, Apa13-2, and Al152-2A) adapted to low temperature and challenged with different copper concentrations. Similar MIC-Cu values were observed among studied strains, but growth kinetic parameters revealed that strain List2-2 was the least affected by the presence of copper at 8°C. This strain was selected for a global transcriptional response study after a 1 h exposition to 0.5 mM of CuSO_4_ × 5H_2_O at 8 and 37°C. The results showed that *L. monocytogenes* apparently decreases its metabolism in response to copper, and this reduction is greater at 8°C than at 37°C. The most affected metabolic pathways were carbohydrates, lipids and nucleotides synthesis. Finally, 15 genes were selected to evaluate the conservation of the transcriptional response in the other two strains. Results indicated that only genes related to copper homeostasis showed a high degree of conservation between the strains studied, suggesting that a low number of genes is implicated in the response to copper stress in *L. monocytogenes*. These results contribute to the understanding of the molecular mechanisms used by bacteria to overcome a combination of stresses. This study concluded that the application of copper in low concentrations in cold environments may help to control foodborne pathogens as *L. monocytogenes* in the industry.

## Introduction

*Listeria monocytogenes* is a Gram positive, non-spore forming, and ubiquitous microorganism which contaminates food and has been frequently associated to foodborne disease (Farber and Peterkin, 1991; Orsi and Wiedmann, 2016). In humans, *L. monocytogenes* can cause listeriosis, a disease that ranges from febrile gastroenteritis to a more severe, invasive disease such as meningitis and septicemia (Franciosa et al., 2005; Lomonaco et al., 2015). This microorganism can grow in different environments such as soil, surfaces, and in a wide variety of food products (Sauder et al., 2012; Zhang et al., 2012; Catwright et al., 2013; Montero et al., 2015). To reduce the microbiological contamination of foods, the industry has developed different strategies. One of the most used is to maintain low temperatures in food processing environments and during final products storage. However, this strategy is not appropriate to control *L. monocytogenes* since this microorganism adapts and grow at low temperatures (Durack et al., 2013; Pittman et al., 2014; Cordero et al., 2016). The food industry applies a combination of stressors –approach known as hurdle technology – to prevent contamination with foodborne pathogens at different stages of the food production (Magalhães et al., 2016). However, it has been also reported that *L. monocytogenes* developed tolerance to different stressors when grown at low temperature (Beales, 2004). For instance, one study showed that cross-resistance to alkali was induced by culturing *L. monocytogenes* strains at low temperatures (Abeysundara et al., 2019). Therefore, new strategies need to be developed to control this foodborne pathogen in the food industry.

Copper, a metal with antimicrobial properties, has the potential to be used as an effective alternative to control *L. monocytogenes* in foods environments. The Environmental Protection Agency of the United States (EPA) acknowledged copper’s antimicrobial properties in 2008 and approved their use as an antimicrobial agent for contact surfaces “in hospitals, other healthcare facilities, laboratories, and various public, commercial, and residential buildings” (EPA, 2008). Several studies have demonstrated copper’s efficiency in reducing the microbial survival of *Escherichia coli O157, Salmonella enterica, L. monocytogenes*, among others (Wilks et al., 2005; Zhu et al., 2012; Parra et al., 2018). Copper toxicity is mediated through different mechanisms, including oxidative stress caused by the generation of toxic reactive oxygen species, formation of adventitious Cu(I)-thiolate bonds, and displacement of iron by copper in the iron-sulfur cluster inactivating essential enzymes (Macomber and Imlay, 2009; Solioz et al., 2010; Ladomersky and Petris, 2015).

Bacterial transcriptional response to copper stress has been widely studied in models such as *Staphylococcus aureus, E. coli*, and *Enterococcus faecalis* (Yamamoto and Ishihama, 2005; Baker et al., 2010; Abrantes et al., 2011). These reports showed that copper not only regulates the gene expression of elements related to copper homeostasis, but it also can trigger different molecular strategies for managing this stress. For example, copper can inhibit nucleotide biosynthesis, modify virulence genes expression, reduce biofilm formation, or activate genes involved in the general stress response (Ward et al., 2008; Baker et al., 2010; Lu et al., 2013).

In a previous study, *L. monocytogenes* was grown at 8 and 30°C and then cultures were challenged with different copper concentrations. As a result, bacterial growth rate declined while copper concentration in the media increased, and this effect was greater at 8°C than at 30°C. Moreover, it was observed that *L. monocytogenes* cultured at 8°C accumulated more copper than same strain cultured at 30°C. These results suggest that there is a synergistic effect between both stressing agents, and that the combination copper/low temperatures might work as a new alternative for the control of *L. monocytogenes* (Latorre et al., 2015).

Understanding the transcriptional response of *L. monocytogenes* to low temperatures and a copper stress could be relevant to develop new strategies to control the pathogen. The aim of this work was to characterize the response of different strains of *L. monocytogenes* simultaneously grown at low temperature and exposed to copper. Furthermore, this work also investigated the bacterial global transcriptional response to an acute copper stress when *L. monocytogenes* was adapted to low temperatures. This approach allowed us to better understand how *L. monocytogenes* transcriptional machinery is modified in response to a combination of stresses.

## Materials and Methods

### Strains and Culture Conditions for Cold Adaptation

Three *L. monocytogenes* strains, isolated from different food matrices were included in the study: List2-2 (seafood), APA13-2 (poultry meat), and Al152-2A (fruit). Isolates were confirmed as *L. monocytogenes* by a PCR described by Bubert et al. (1992). For low temperature assays, bacteria were adapted in a two-step process: the first day, a single colony was inoculated into Tripticase Soy Broth (BBL, Becton Dickinson, United States) containing 0.6% yeast extract (Oxoid, Basingstoke, United Kingdom; TSBYE) and cultured at 37°C overnight at 160 rpm. The next day, a cold, fresh TSBYE broth was inoculated with *L. monocytogenes* and adjusted to 600 nm (OD_600nm_): 0.05. The culture was grown at 8°C (160 rpm) for 72 h. For assays at 37°C, a *L. monocytogenes* single colony was incubated at in TSBYE at 37°C (160 rpm) overnight. All the microbiological assays were performed in a biosafety level II-approved laboratory accessible only to trained research individuals working with human foodborne pathogens.

### Serotyping

*Listeria monocytogenes* strains were serotyped by a multiplex PCR according to the protocol and primers described by Doumith et al. (2004).

### Determination of the Minimum Inhibitory Concentration of Copper (MIC-Cu)

In order to evaluate the MIC-Cu, the broth dilution method was used (Andrews, 2001). For the assay, TSBYE was supplemented with different copper concentrations (CuSO_4_ × 5H_2_O: 2, 4, 6, 8, 10, 12, and 14 mM) and then inoculated with 1 × 10^5^ *L. monocytogenes* CFU/mL. For assays at 8°C, strains were adapted at low temperature as described above. Assays were performed at 8°C for 10 days or at 37°C for 24 h, under agitation at 160 rpm. MIC-Cu was defined as the lowest copper concentration at which no bacterial growth was observed. All experiments were performed in triplicates, and control cultures were performed by not adding copper to the culture media.

### Copper Cell Content Measurement

Intracellular copper content was determined in bacteria adapted to low temperature (8°C) or cultured at 37°C after 1 h exposure to 0.5 mM CuSO_4_ × 5H_2_O. Samples were processed as described by Latorre et al. (2015). Samples were measured with a S2 PICOFOX^TM^ TXRF spectrometer (Bruker, Germany) (González et al., 1999; Fiedor et al., 2016).

### Growth Curves

The effect of copper on *L. monocytogenes* growth was determined at two different copper concentrations. Assays were run in TSBYE supplemented with 2 and 3 mM CuSO_4_ × 5H_2_O, and negative control were included (not copper added). TSBYE tubes were inoculated at OD_600nm_: 0.05 with different *L. monocytogene*s strains and cultured at 8 or 37°C under agitation (160 rpm). For experiments at 8°C, bacteria were adapted to low temperature as described above, and growth was controlled by measuring OD_600nm_ twice a day for 5 days. For assays at 37°C, growth was controlled by measuring OD_600nm_ every hour for 8 h. All growth curve experiments were performed in triplicates.

### Microarray Experiments

Strain List2-2 was selected for global expression experiments. Four experimental conditions were tested: (i) 8°C, (ii) 8°C+0.5 mM CuSO_4_ × 5H_2_O, (iii) 37°C, and (iv) 37°C + 0.5 mM CuSO_4_ × 5H_2_O. For experiments at low temperature, fresh TSBYE tubes at 8°C were inoculated with *L. monocytogenes* adapted to low temperature. Cultures were adjusted to OD_600nm_: 0.05. For experiments at 37°C, List2-2 was grown overnight and then transferred to fresh TSBYE media and adjusted to OD_600nm_: 0.05. When cultures reached OD_600nm_: 0.5, strains were challenged with 0.5 mM of copper for 1 h. After copper exposure, bacterial cells were collected, and total RNA was extracted using the RNeasy Kit (Qiagen). RNA concentration was measured with a NanoDrop ND-1000 instrument, and RNA integrity was evaluated in a gel electrophoresis.

cDNA was synthesized and labeled with Cy3 (green) fluorescent dye (GE Health Care) following manufacturer instructions. Labeled cDNA was hybridized to slides according to Microarrays, Inc. recommendations. Each slide contained 70-mer probes representing 2,857 ORF of *L. monocytogenes* strain EDG-e in triplicates. Slides were digitalized using an Agilent technologies scanner, and signal intensities were measured using the program Scan Array Express (Perkin Elmer). Microarray data are available in Supplementary Table S2.

### qPCR

qPCR assays were conducted using strains List2-2, APA13-2, and Al152-2A. Bacteria were cultured under the same conditions described for the microarray assays, and the same RNA extraction protocol was used. The cDNA synthesis, primers design (Supplementary Table S1), and the transcripts level quantification were done as previously described by Cordero et al. (2016).

### Statistical Analysis

All data was analyzed using the R studio software (RStudio Team, 2016). The *GrowthcurveR-package* was used, and data was fitted to a logistic model to analyze bacterial growth dynamics (Sprouffske and Wagner, 2016). Microarray analysis data were normalized using the quantile method (Bolstad et al., 2003). To analyze spot intensities data, a Student *t*-test recommended by the *Limma* protocol was used. It was considered that a gene was differentially expressed when the log-fold change was greater than 1 or below –1 (*P*-value < 0.05). Using a Kruskal–Wallis test genes with differentially expression in a qPCR analysis were identified. Also, a Dunn test was used as a *post hoc* analysis among strains with differences statistically significant. Genes were considered statistically significant if their *P*-value were less than 0.05.

## Results and Discussion

### Effect of Copper on the Growth of *L. monocytogenes* at Low Temperature

Previously characterized *L. monocytogenes* strains (*n* = 3) were selected based on their growth rate at low temperature (Cordero et al., 2016). Selected strains displayed a fast-growing rate at 8°C. These strains were isolated from different foods, and they presented the most frequently reported *L. monocytogenes* serotypes in Chile (List 2-2: 1/2a; APA13-2: 4b; and AL152-2A: 1/2b) (Montero et al., 2015). According to these results, strains in this study are potentially pathogenic since these serotypes are linked to 95% of infections caused by *L. monocytogenes* in humans (Catwright et al., 2013; Montero et al., 2015). These findings show that *L. monocytogenes* contaminating foods in Chile represent a risk to public health, and that surveillance activities might be needed to evaluate potential impact to the population.

The tolerance of three strains to copper at two temperatures was evaluated (8 and 37°C). MIC-Cu results showed that *L. monocytogenes* is more tolerant to copper at 37°C (10–12 mM) than at 8°C (4–6 mM). These observations suggest that cold increases the toxic effect of copper on *L. monocytogenes.* This corroborates previous observations by Latorre et al. (2015). In previous studies, a similar MIC-Cu value (MIC-Cu 12 mM) was reported for *L. monocytogenes* strain 2011L-2858 cultured at 37°C. The strain was serotype 1/2b, isolated in 2011 from melon and related to a foodborne outbreak in the United States (Parsons et al., 2017).

**FIGURE 1:**
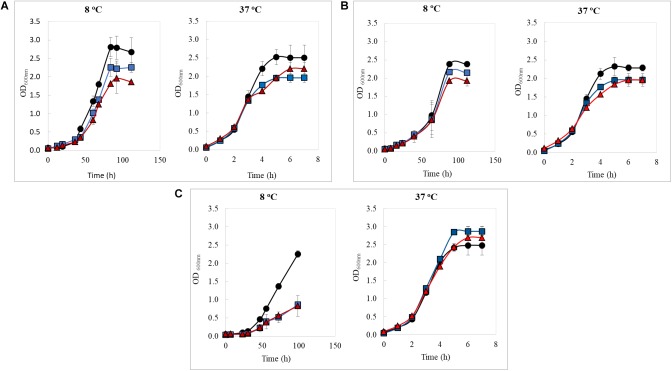
Growth curves of *Listeria monocytogenes* strains at 8 and 37°C in TSBYE media supplemented with copper (blue line: 2 mM CuSO_4_ × 5H_2_O; red line: 3 mM CuSO_4_ × 5H_2_O, and black line: control not copper added). **(A)** List2-2, **(B)** APA13-2, **(C)** AL152-2A. All values represent the average of three biological replicates in triplicates. Error bars designate standard deviation (*n* = 9).

The kinetic growth of the three strains in study was evaluated in the presence of a non-toxic copper concentrations (2 and 3 mM of CuSO_4_ × 5H_2_O) at 8 and 37°C (Figure 1). Growth data were adjusted to a logistic model, and the maximum bacterial growth (K) and generation time (Gt) parameters were obtained to compare different growing conditions as presented in Table 1. At 8°C, *K* parameter reached for all three strains decreased when the copper concentration increased; however, at 37°C, changes in *K* values seem independent from copper concentration tested. It was also observed that bacterial Gt increased as the copper in the medium at both temperatures; List2-2 was least affected by copper at 8°C, while Al152-2A increased its Gt by 50% when compared to the control at 2 mM copper. These results indicate that List2-2 was the more tolerant strain to copper when growing at low temperature.

**Table 1:** Kinetic parameters of *Listeria monocytogenes* strains growing in environment supplemented with different copper concentrations at 8 and 37°C.

ID Strains	Copper Concentration	K^•^ (OD600 nm max)		Standard error ^#^ (K)		Gt ^Δ^ (h)		Sigma^♢^	
	(mM)	8°C	37°C	8°C	37°C	8°C	37°C	8°C	37°C
List2-2	0	2.79	2.54	0.21	0.03	6.84	0.45	0.12	0.06
	2	2.32	1.92	0.14	0.05	7.21	0.44	0.09	0.03
	3	1.91	2.19	0.17	0.11	6.83	0.66	0.07	0.11
APA13-2	0	2.48	2.25	0.18	0.04	7.70	0.39	0.15	0.04
	2	2.29	1.92	0.17	0.06	8.94	0.45	0.16	0.03
	3	2.05	1.86	0.12	0.03	9.39	0.59	0.12	0.04
Al152-2A	0	2.53	2.44	0.12	0.03	9.9	0.44	0.09	0.03
	2	1.56	2.90	0.11	0.06	14.83	0.50	0.10	0.08
	3	1.48	2.66	0.18	0.03	14.79	0.57	0.19	0.04


*^∗^Kinetic parameters were obtained from the proliferation curves showed in Figure 1; ^∗∗^Experimental conditions: Incubation time was 112 h when cultures were incubated at 8°C and 8 h when incubation was performed at 37°C; ^•^K: Estimation of the Maximum bacterial growth measured at 600 nm (OD600 nm max) as calculated from the logistic model; All *K* values were statistically significant (*p* < 0.05). ^#^Standards error of the *K* parameter; ΔGt (h): generation time (in hours) calculated under each condition; ♢ Sigma: Residual standard error from non-linear least squares fit of the model to the data; Values near to 0 have a better fit to the model.*

Previous studies have reported that variations in microbial proliferation rates are related to greater levels of gene-expression (Klumpp and Hwa, 2008; Gerosa et al., 2013; Borkowski et al., 2015). In fact, Borkowski et al. (2015), using fast and slow growing *Bacillus subtilis* strains, showed that the abundance of total RNA is proportional to the bacterial proliferation rate. This correlation has been discussed over time (Warringer et al., 2003; Feder and Walser, 2005), but recently Jensen et al. (2017) demonstrated a link between fitness and gene expression changes in *Streptococcus pneumoniae* through genome-wide metabolic models. Considering that growth behavior differed among *L. monocytogenes* strains in the presence non-toxic concentrations of copper, it is possible that List2-2 may have a different transcriptional response to copper at low temperature than the other two strains in the study. Other possible explanations for different growth rates could be the formation of adventitious bonds between copper and key sites in proteins, altering enzymatic functions and causing negative effects in bacterial growth, as seen in *E. coli* by other authors (Stewart et al., 2019). Results obtained in this study suggest that adaptive mechanisms of *L. monocytogenes* exposed to copper are strain dependent and also vary depending on the temperature. Therefore, considering that List2-2 was more tolerant to the combination low temperature-copper, it is interesting to learn how this strain respond to both stresses at a transcriptional level.

### Global Transcriptional Response of *L. monocytogenes* List2-2 to Copper Acute Stress

In order to evaluate the global transcriptional response of *L. monocytogenes* to an acute, non-lethal copper exposure (0.5 mM), microarray assays were carried out for List2-2 strain incubated at two temperatures (8 and 37°C). This concentration was the highest tested that did not affect bacterial kinetics when considering the three chosen strains, and it is equivalent to 1/8 of the MIC-Cu. These data were obtained in the experimental set-up (data non-shown).

**FIGURE 2:**
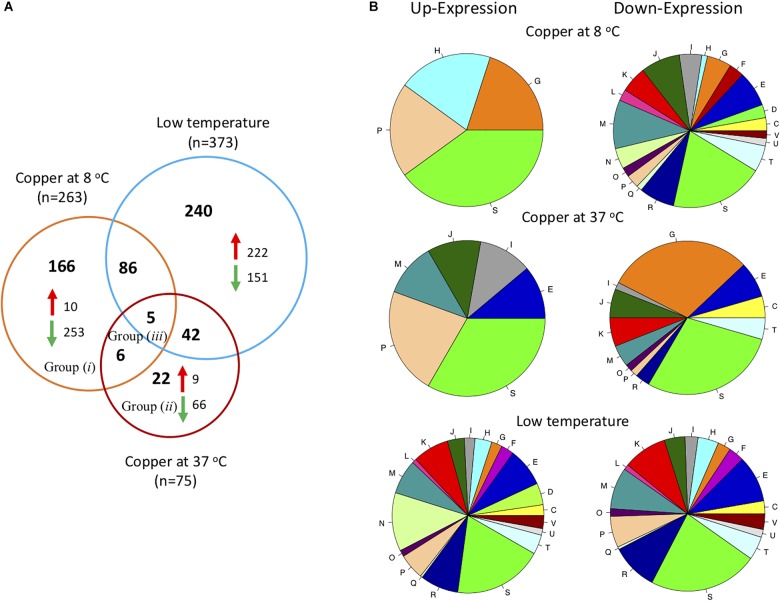
Analysis of transcriptional response of *L. monocytogenes* List2-2. **(A)** Venn diagram showing the differentially expressed genes in List2-2 challenged with copper at 8 and 37°C and differentially expressed genes at low temperature (8°C). **(B)** Differentially expressed genes in response to copper at two temperatures grouped by COGs: **C:** Energy production and conversion; **D:** Cell cycle control, cell division, chromosome partitioning; **E:** Amino acid transport and metabolism; **F:** Nucleotide transport and metabolism; **G:** Carbohydrate transport and metabolism; **H:** Coenzyme transport and metabolism; **I:** Lipid transport and metabolism; **J:** Translation, ribosomal structure and biogenesis; **K:** Transcription; **L:** Replication, recombination and repair; **M:** Cell wall/membrane/envelope biogenesis; **N:** Cell motility; **O:** Post-translational modification, protein turnover, chaperones; **P:** Inorganic ion transport and metabolism; **Q:** Secondary metabolite biosynthesis, transport and catabolism; **R:** General function prediction only; **S:** Function unknown; **T:** Signal transduction mechanisms; **U:** Intracellular trafficking, secretion, and vesicular transport; **V:** Defense mechanisms.

To select genes that changed their expression by the combined effect of low temperature and copper, it was necessary to determine first the genes that were affected by low temperature. For this, the transcriptome of *L. monocytogenes* grown at 37°C was compared to the transcriptome of those cultured at 8C. It was observed that 373 genes (approximately 13% of *L. monocytogenes* genome) changed their expression by low temperature (Figure 2A); out of those, 240 can be explained by low temperature only, and 133 other genes changed their expression when copper is added (at 37°C and at 8°C).

Then, the response to copper at 37°C was examined to compare and identify the effects of copper at optimal growth temperature. These results showed that 75 genes modified their expression in response to copper at 37°C; nine genes were up-regulated, and 66 genes were down-regulated (Figure 2A). The effect of copper over the global transcriptional response has been studied in other bacterial models. Quintana et al. (2017) reported that *Pseudomona aeruginosa* gene expression was modified after exposure to copper for 5 min at 37°C. Out of the 531 genes that changed, 71% were down-regulated. However, this response was reverted when cultures were exposed to the metal for longer times (2 h), and 70% of genes up-regulated. The later response comprised genes related to copper homeostasis and genes involved in other metabolic pathways required for bacterial growth. Yamamoto and Ishihama (2005) evaluated the transcriptional response of *E. coli* exposed to 0.5 mM of copper at 37°C. These authors found that 37 of 38 genes (97.4%) were over-expressed after 5 min of exposure to copper. Over-expressed genes were those participating in copper homeostasis: *copA* (metal output transporter), *cueO* (multicopper oxidase), and *cusC* (copper pump component). These results suggest that differences in transcriptional response to copper seem to be species-dependent.

Genes that were up-regulated in *L. monocytogenes* challenged with copper at 37°C were mainly related to copper homeostasis previously identified by Corbett et al. (2011): lmo1854: transcriptional regulator CsoR; lmo1853: P1-type ATPase CopA; lmo1852: copper metallochaperone CopZ. On the contrary, down-regulated genes were primarily involved in carbohydrate transport and metabolism (*n* = 20; COG class G) such as genes that encode the phosphotransferase system (PTS; genes lmo0027, lmo0298, lmo0299, lmo1997, lmo2000, lmo2001, lmo2124, lmo2125, lmo2665, lmo2666 and lmo2708) (Figure 2B and Supplementary Table S2). The effect of copper over the carbohydrate metabolism has been described in other bacterial models. When a *E. faecalis* *cop* operon mutant (*cop*Δ mutant) was exposed to copper at 37°C, the bacteria down-regulated genes coding the ABC transporters which are linked to sugar uptake (Latorre et al., 2014). The authors suggest that sugar uptake transporters are related, directly or indirectly, to mechanisms linked to copper uptake. To date, bacterial mechanisms linked to copper uptake have not been described. Therefore, it is possible to hypothesize that sugar transporters may also transport copper and other molecules in an unspecific manner. This is supported by observations showing that *L. monocytogenes* down regulates other sugar transporters (man-PTS regulators) to avoid bacteriocins negative effects (Gravesen et al., 2002; Balay et al., 2018).

In this study, when the *L. monocytogenes* was cultured at low temperature (8°C) and exposed to copper, a total of 263 genes were differentially expressed; out of those, 34% (91 genes) also changed when bacteria were exposed to low temperature as a unique stress factor, but only 11 showed differences when *L. monocytogenes* was exposed to copper at 37°C (Figure 2A). *copZA* operon genes were among the few (*n* = 10) that were up-regulated when List2-2 was exposed to copper and low temperature simultaneously. On the other hand, most genes were down-regulated (96.2%) under the same condition. Genes involved in membrane biogenesis (COG class M) were the most affected by the experimental conditions: 26/103 class M genes were repressed (Figure 2B). Previous studies indicated that *L. monocytogenes* modify its membrane composition in response to low temperature. This is achieved by modifying the expression of genes related to membrane synthesis (Zhu et al., 2005).

Further, when comparing the number of genes that change their expression levels among the different experimental conditions, a larger number of genes changed when more stress factors were applied to bacteria. For instance, a larger number of genes changed their expression when *L. monocytogenes* was exposed to copper and low temperature than when bacteria were challenged to copper as a unique stressing factor. It was also observed that the transcriptional response to copper -at 8°C and at 37°C- involved fewer genes than when *L. monocytogenes* was exposed only to low temperature. These results suggest that the stress caused by the combination of low temperature and copper triggers a different response than when bacteria are challenged by each stimulus separately, and that *L. monocytogenes* may deflect its metabolism toward functions directly related to handling this “new” stress condition.

Global expression results were integrated into a metabolic network to identify the main pathways that were affected in *L. monocytogenes* by an acute copper exposure (Supplementary Figure S1). Results showed that, at low temperature, copper decreases the expression of genes encoding for proteins involved in pathways related to nucleotide, carbohydrate, and lipid synthesis. At 37°C, these pathways were less affected and fewer genes were down-regulated. Consequently, we inferred that the general metabolism is more affected by copper exposure at low temperature than at 37°C. Moreover, Johnson et al. (2015) reported that *S. pneumoniae* nucleotide synthesis was inhibited when the culture was exposed to copper (200 μM, 37°C), similarly to what was observed in this study (Supplementary Figure S1). Since nucleotides play an important role in the regulation of cellular processes (Kilstrup et al., 2005), it can be suggested that the reduction in the proliferation rate in List2-2 may be the result of the effect of copper and cold stresses over the nucleotide synthesis (Figure 1).

### Specific Transcriptional Response to Acute Copper Stress in *L. monocytogenes* Strains

**FIGURE 3:**
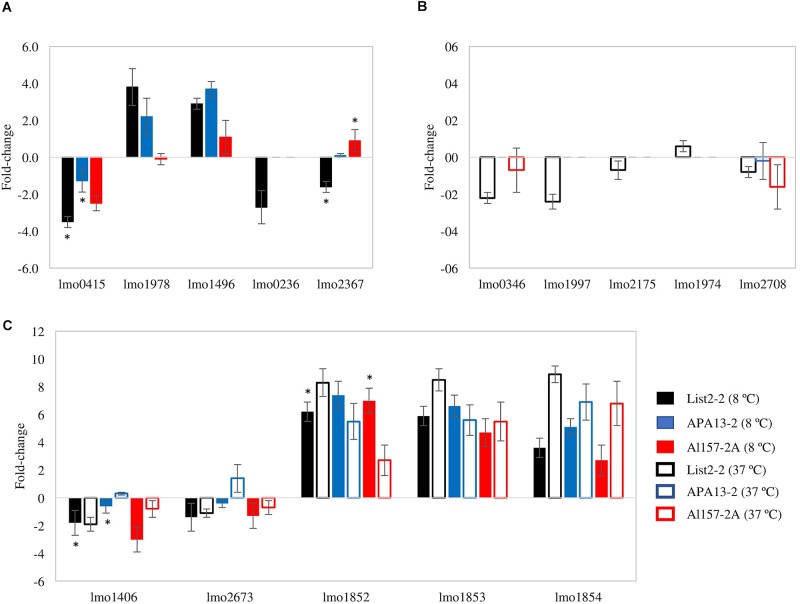
Fold changes of *L. monocytogenes* strains in response to copper. **(A)** fold change at 8°C (Figure 2A, Group *i*). **(B)** fold change at 37°C (Figure 2A, Group *ii*). **(C)** fold change at both temperatures (Figure 2A, Group *iii*). All values represent the average of two replicates and error bars denotes standard deviation. ^∗^ represent significant differences in the expression of the specific gene between strains (*P*-value < 0.05).

In order to validate the observations from the global transcriptional analysis, and to compare the response of List2-2 to other strains, the expression levels of 15 genes of *L. monocytogenes* strains APA13-2 and Al152-2A were evaluated by qPCR. Genes were selected based on microarray results (Figure 2A: Group *i*: copper effect at 8°C; Group *ii*: copper effect at both temperatures; and Group *iii*: copper effect at 37°C). From each identified group, 5 genes were selected. Only five of fifteen genes exhibited a similar expression level in all three strains evaluated. Among those, the three genes encoded in the *copZA* operon were significantly up-regulated when bacteria were exposed to an acute copper stress at both, 8 and 37°C (Figure 3). Among the other 10 genes evaluated, six genes –related to carbohydrate and lipid metabolism– showed a different response between List2-2 and the other two strains studied. Previous studies have reported differential gene expression in *L. monocytogenes* strains exposed to stress (Rantsiou et al., 2012; Cabrita et al., 2015). Cabrita et al. (2015) identified different gene expression profiles for nine stress related genes in sporadic and persistent strains exposed to cold, and this was associated to different gene expression networks in each *L. monocytogenes* strain. In this study, differences observed at the transcriptional level may explain the fluctuations at the phenotypic level among *L. monocytogenes* strains. Moreover, signals for other four genes were not detected (lmo0236, lmo1974, lmo1997 and lmo2175) in these two strains. Genomic information recently obtained demonstrated that those genes are not present in APA13-2 or Al152-2A strains (data not shown).

**FIGURE 4:**
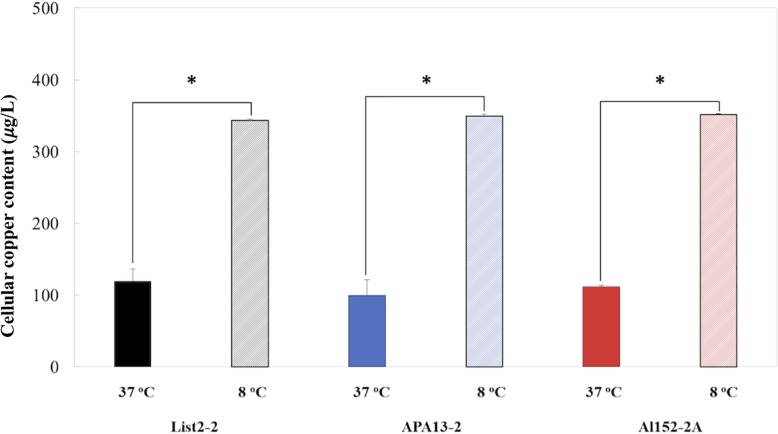
Intracellular copper content of *L. monocytogenes* strains cultured at 37 and 8°C after 1 h exposure to 0.5 mM of CuSO_4_ × H_2_O. All values are expressed as the direct copper content normalized by the colony-forming unit (1 × 10^8^). All values represent the average of three replicates and error bars denotes standard deviation. All values represent the average of three replicates and error bars denotes standard deviation. ^∗^represent significant differences between pairs (*P-*value < 0.05).

To better understand how *L. monocytogenes* manages copper, the intracellular copper content was measured after an acute copper stress (0.5 mM of CuSO_4_ × 5H_2_O × 1 h) in all three strains at both tested temperatures (8 and 37°C). It was observed that copper intracellular content did not differ among strains grown at same temperature, but all *L. monocytogenes* strains accumulated near four times more copper at 8°C than at 37°C (Figure 4). On the contrary, *cop*A (heavy metal-transporting ATPase) transcription levels were similar in both temperatures tested. This discrepancy between expression levels and the intracellular copper content might be due to the activation of some post-transcriptional mechanisms that may be affecting transcript stability at 8°C, consequently reducing the translation of those proteins (Horn et al., 2007; Saberi et al., 2016; Jackson et al., 2017). These mechanisms may explain differences in intracellular copper content observed when growing *L. monocytogenes* at different temperatures.

In summary, it was observed that the main effect of sub-inhibitory concentrations of copper in *L. monocytogenes* cultured at low temperature was a reduction in bacterial growth rate. This may be probably related to the deleterious effect of copper over *L.* *monocytogenes* metabolism. The transcriptional analysis of *L. monocytogenes* exposed to 1 h to copper resulted in a marked gene down-regulation. This suggests that *L. monocytogenes* could be making an additional effort to remain viable under the combination of these stressful conditions. In addition, it was found that the transcriptional response to copper was conserved for genes directly related to copper homeostasis, but it was not conserved for genes involved in other cell functions independently of temperatures evaluated. Finally, the study of the transcriptional response in *L. monocytogenes* gave us information on the possible metabolic pathways most affected by the combination of cold and copper stresses.

The experimental approach used helped to comprehend how this foodborne pathogen might behave in real environments where these stressors (low temperature and copper) occur simultaneously. It was also possible to understand how *L. monocytogenes* adjusts its transcriptional machinery to survive and proliferate in adverse environments, such as the combined stresses. However, it has yet to be elucidated why different *L. monocytogenes* strains respond to the stress combination in an individual fashion. To answer some of these questions it is necessary to study molecular mechanisms that define these phenotypic differences. Understanding the transcriptional networks activated when *L. monocytogenes* is under these stressors could also help understand how the bacteria adapts to copper and how copper could be used for controlling this foodborne pathogen.

Finally, results in the present study suggest that copper may be used in the food industry. Copper has been used as an antimicrobial agent since ancient times (Grass et al., 2011). However, in high concentrations, copper could be toxic for human beings (Gaetke and Chow, 2003). This highlights that the use of copper in the food industry should only consider non-food contact surfaces such as ceilings, floors, walls, etc. This implementation could reduce the contamination of surfaces with *L. monocytogenes*, and consequently, it may have an impact reducing the risk of cross-contamination of food products. Further studies measuring the transfer rate from copper surfaces to different foodstuff could shed light on the potential use copper on food contact surfaces.

## Author Contributions

AQ-V, AR-J, and MG designed the research. AQ-V, AP, and FM performed the research. AQ-V, AP, and AR-J analyzed the data. AQ-V, AP, PN, MG, ML, MT, and AR-J wrote the manuscript.

## Conflict of Interest Statement

The authors declare that the research was conducted in the absence of any commercial or financial relationships that could be construed as a potential conflict of interest.

## Supplementary Material

FIGURE S1 Metabolic pathway activated in response to copper in List2-2 strain. **(A)** low temperature (8°C) and **(B)** 37°C. Lines in color represents putative pathway activated during copper exposure. Green down regulated genes, red up-regulated genes. Metabolic network was built using the program IPath 3.0.

TABLE S1 Sequences of primers used in the study.

TABLE S2 Microarray data analysis. Green represents down-regulated genes and red represents up-regulated genes.
