# Supplementary figures and images for: The Combined Effect of Cold and Copper Stresses on the Proliferation and Transcriptional Response of Listeria monocytogenes
Source: Front Microbiol. 2019 Mar 28;10:612. doi: 10.3389/fmicb.2019.00612 (PMC6447683; doi:10.3389/fmicb.2019.00612)

## Slide 1
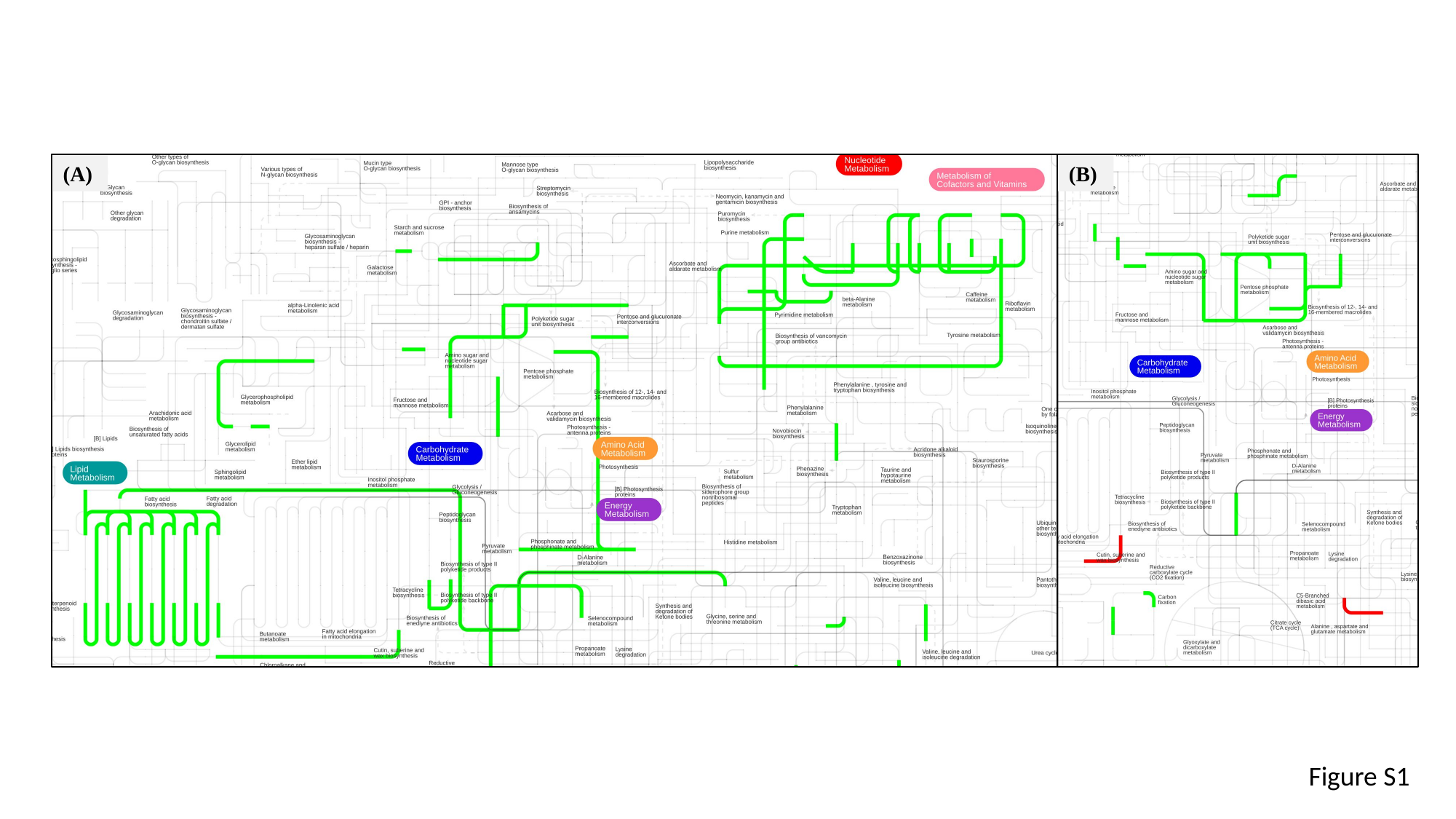

(A)
(B)
Figure S1

Supplement: FIGURE S1 — Metabolic pathway activated in response to copper in List2-2 strain. (A) low temperature (8°C) and (B) 37°C. Lines in color represents putative pathway activated during copper exposure. Green down regulated genes, red up-regulated genes. Metabolic network was built using the program IPath 3.0. [file Presentation_1.PPTX]
